# Comparative Genomics of *NGF*, *NTRK1*, and *NGFR* Across Chordates: Joint Gene Recovery and Region-Specific Constraint

**DOI:** 10.3390/biology15191695

**Published:** 2026-09-23

**Authors:** Zhimin Li, Xiaojie Liu, Ya Zhang, Yeping Tan

**Affiliations:** 1 Institute of Veterinary Medicine, Jiangsu Academy of Agricultural Sciences, Key Laboratory of Veterinary Biologicals Engineering and Technology, Ministry of Agriculture and Rural Affairs, National Center for Engineering Research of Veterinary Bio-Products, Nanjing 210014, China; lizhimin1989@hotmail.com (Z.L.); ya_zhang09@foxmail.com (Y.Z.); 2 School of Marine Science and Engineering, Nanjing Normal University, Nanjing 210023, China; liuxiaojieyx@hotmail.com

**Keywords:** nerve growth factor, neurotrophin, TrkA, p75NTR, comparative genomics, orthology, chordates, gene recovery, purifying selection, structural constraint

## Abstract

Nerve growth factor (NGF) is a neurotrophin central to sensory neuron biology and pain signaling. *NGF* and *NTRK1* are gnathostome paralogs within the older neurotrophin and Trk families, respectively, whereas *NGFR* encodes p75NTR, a member of the tumor necrosis factor receptor superfamily. Orthology assessment of *NGF*, *NTRK1*, and *NGFR* across vertebrates is challenging because mature neurotrophins are short, cysteine-rich ligands, *NTRK1* has closely related paralogs, and *NGFR* lies outside the Trk receptor family. We assessed species-level joint genomic recovery of the three genes and compared evolutionary constraint among predefined ligand and receptor regions. The analysis covered 94 chordates and combined graded recovery evidence with family phylogenies, locus and domain evidence, codon models, amino acid conservation, and structural mapping. Sixteen non-gnathostome species were included to provide evolutionary context and were not used to calculate recovery rates for the three focal genes. Among 78 sampled gnathostomes, 52 had high-confidence joint recovery, eight had supported provisional joint recovery, four had component-level evidence consistent with possible co-recovery, and 14 remained incomplete. Mature NGF had a numerically lower estimate of the ratio of nonsynonymous to synonymous substitution rates (dN/dS, ω) than the pro-domain (0.067 versus 0.352). All six scaffold cysteines were invariant; in the residue-class comparison, the cysteine scaffold was the only structural class that differed from the coordinate-resolved background after correction. The dimer-interface, TrkA-contact, and p75NTR-contact classes did not differ significantly from that background after correction. Receptor analyses yielded low ω estimates in Trk kinase domains and p75NTR intracellular regions. Together, these results provide a confidence-graded map of focal-gene recovery and regional purifying constraint while placing non-gnathostome homologs within the older neurotrophin system.

## Simple Summary

Nerve growth factor (NGF) promotes survival of specific nerve cells, including those involved in sensing pain and touch, and also contributes to pain and several diseases. It signals mainly through TrkA, encoded by *NTRK1*, while p75NTR, encoded by *NGFR*, can modify TrkA signaling or mediate distinct responses. We examined whether the genes for *NGF*, *NTRK1*, and *NGFR* could be identified together in available genomic data from 94 chordate species, including fishes, amphibians, reptiles, birds, and mammals. Early-diverging chordates and cyclostomes provided evolutionary context rather than gene absences, since they split from the gnathostome lineage before the ancestral neurotrophin system gave rise to the named *NGF* and *NTRK1* genes. We combined information from gene sequences, nearby genes, protein features, and evolutionary comparisons. Among 78 sampled jawed vertebrates, 52 had high-confidence identification of all three genes, eight had supported provisional identification, four had only partial evidence, and 14 remained incomplete. Mature NGF was highly conserved, especially six cysteines that help maintain its shape, and internal regions of the receptor proteins were also strongly conserved.

## 1. Introduction

Nerve growth factor (NGF) is the founding neurotrophin and one of the best characterized secreted regulators of neuronal survival, sensory neurobiology, and pain signaling [1,2,3,4]. NGF signals primarily through TrkA (encoded by *NTRK1*), whereas p75NTR (encoded by *NGFR*) can modulate NGF-TrkA binding and downstream signaling or participate in distinct receptor contexts [3,5,6,7]. Beyond these canonical neural roles, NGF signaling has been implicated in neural ingrowth after injury, skeletal repair, osteoarthritic remodeling, tendon fibrosis, and reduced response to lenvatinib in hepatocellular carcinoma [8,9,10,11,12]. These diverse biological roles make it useful to examine both the genomic plasticity of the three genes and the distribution of evolutionary constraint across ligand and receptor regions.

NGF is synthesized as preproNGF. Signal-peptide removal produces proNGF, and proteolytic processing releases mature NGF; proNGF can also elicit responses distinct from those of the mature ligand [7,13,14]. At the molecular level, mature NGF forms a homodimer with a cystine-knot fold. Structures of NGF complexes with TrkA and p75NTR define the dimer interface and receptor-contact surfaces [5,15,16]. The receptors also differ in domain architecture: TrkA contains extracellular leucine-rich and immunoglobulin-like domains and an intracellular tyrosine kinase domain, whereas p75NTR contains extracellular cysteine-rich domains and an intracellular death domain [6,17,18]. These structural features and receptor domains define biologically interpretable regions for comparative analysis. Accordingly, *NGF*, *NTRK1*, and *NGFR* were treated as the focal gene set for comparative genomic analysis.

Previous studies have reconstructed the diversification of the neurotrophin signaling system [19,20,21,22,23,24,25]. Yet, their focus was mainly on gene-family evolution rather than species-level recovery confidence. Indeed, the neurotrophin signaling system predates the named gnathostome paralogs examined here. An amphioxus Trk-family receptor has been experimentally characterized, lampreys retain neurotrophin-family homologs, and loss of Trk-family components has been proposed in *Ciona* [26,27,28]. Studies in selected protostomes also identified homologs of neurotrophins, Trk receptors, and p75NTR within the same species [29,30], but those were limited to individual taxa or gene-family histories. It remains unclear how consistently the specific gnathostome genes *NGF*, *NTRK1*, and *NGFR* can be recovered together across a broad chordate sample and how the confidence of these assignments varies among clades. Species-level recovery confidence and regional constraint in the ligands and receptors have generally been analyzed separately. Species-level assignment requires more than sequence similarity within a single gene family because mature neurotrophins are short cysteine-rich ligands, *NTRK1* must be distinguished from *NTRK2* and *NTRK3*, and *NGFR* requires separate evaluation outside the Trk family [25,31].

This assignment difficulty is compounded by the fact that early vertebrate genome duplication events generated closely related paralogs within the ancestral neurotrophin and Trk receptor gene families. Recent chromosome-scale reconstructions support a vertebrate-stem duplication before cyclostome–gnathostome divergence, followed by separate gnathostome- and cyclostome-stem polyploidizations; the complex cyclostome rediploidization history further complicates one-to-one orthology assignment [32,33]. Subsequent genome rearrangement, lineage-specific gene retention or loss, and variation in assembly continuity and annotation complicate comparisons among clades [21,32,33,34,35]. These factors complicate discrimination between biological absence and technical non-recovery, particularly in early-diverging vertebrates, where family-level homologs cannot necessarily be assigned one-to-one to the later named *NGF* or *NTRK1* paralogs. Simple presence–absence calls can therefore conceal both assignment uncertainty and gene-family history. Graded, paralog-aware evidence helps distinguish these possibilities across uneven genomic resources. Here, ‘joint genomic recovery’ denotes recovery of *NGF*, *NTRK1*, and *NGFR* from the genomic resources examined for a given species. This genomic category is distinct from evidence of co-expression or an obligatory functional complex. Non-gnathostome species therefore provide an evolutionary boundary for the analysis. Their homologs represent the ancestral neurotrophin system, and these species were not included when calculating recovery rates for the three focal genes.

We analyzed 94 chordate species using graded evidence for gene recovery, family phylogenies with paralog context, locus and domain evidence, codon models, amino acid conservation, and structural mapping. *BDNF*, *NTF3*, *NTF4*, *NTRK2*, and *NTRK3* were included as paralogous context for orthology assessment. We first distinguished high-confidence, provisional, possible, and incomplete joint recovery of *NGF*, *NTRK1*, and *NGFR* and then evaluated evolutionary constraint across NGF precursor regions, structural features of mature NGF, and receptor domains [5,15,16,36]. Together, these analyses characterize species-level genomic recovery and protein-region constraint.

## 2. Materials and Methods

### 2.1. Species Panel and Focal Genes

We analyzed a panel of 94 chordate species comprising 4 cephalochordates, 6 tunicates, 6 cyclostomes, 6 chondrichthyans, 20 ray-finned fishes, 2 non-tetrapod sarcopterygians, 30 non-mammalian tetrapods, and 20 mammals. The gene panel comprised *NGF*, *BDNF*, *NTF3*, *NTF4*, *NTRK1*, *NTRK2*, *NTRK3*, and *NGFR*. *NGF*, *NTRK1*, and *NGFR* were treated as the focal gene set; the other neurotrophins and Trk receptors were included as paralogous context for orthology assessment. Sampling was purposive and intentionally uneven: early-diverging groups defined the boundary between ancestral family-level homologs and named gnathostome paralogs, whereas osteichthyans were sampled more densely for the focal comparisons. Species were prioritized when an identifiable assembly and accession-bearing annotation, GFF record, or comparative locus could be evaluated. Assembly continuity and annotation depth were not modeled as covariates; the reported proportions therefore describe this sampled panel rather than all members of each clade. The 16 cephalochordate, tunicate, and cyclostome species were treated as evolutionary boundary taxa and were not included when calculating joint recovery rates for the three focal genes. Joint-recovery proportions were therefore calculated only for the 78 sampled gnathostomes.

### 2.2. Gene Recovery Inventory

NCBI Datasets gene and ortholog packages were the primary source of accession-bearing gene, transcript, and protein records [37]. UniProt, OrthoDB, and Ensembl comparative annotations [38,39,40] were used for discovery and cross-validation. Strict searches by gene symbol provided an additional annotation source. Each gene-species combination was assigned to one of six operational evidence states: high-confidence focal assignment, synteny-supported candidate, sequence-only candidate, conflicting/rejected hit, not recovered, or not applicable for focal-paralog assignment. High-confidence assignments required an accession-bearing record with concordant annotation and sequence/family evidence and no material phylogenetic, locus, or architecture conflict. Synteny-supported candidates had plausible sequence assignments supported by local synteny, whereas sequence-only candidates lacked sufficient locus continuity or a complete gene model. Conflicting/rejected hits were not counted as recovered. ‘Not recovered’ means that no focal candidate met the present criteria and does not establish biological absence or gene loss. Family-level homologs that could not be mapped one-to-one to named gnathostome paralogs were classified as not applicable. When multiple records were retrieved, they were consolidated into one call per gene per species. Within each accession-bearing gene record, representative transcripts were prioritized by MANE or RefSeq Select status, curated protein accession, protein-coding annotation, and protein length. Additional records from the same sampled taxon remained separate in sequence-level analyses but did not add species-level recovery votes. High-confidence joint recovery required high-confidence assignments for *NGF*, *NTRK1*, and *NGFR*; supported provisional joint recovery allowed synteny-supported candidates but no sequence-only component. Cases containing a sequence-only component were classified as component-level evidence consistent with possible co-recovery. Supplementary Tables S1–S4 provide accessions, evidence states, clade summaries, and analysis-inclusion rules.

Selected proportions were summarized with Wilson 95% confidence intervals. A total of 4 focused exploratory contrasts used two-sided Fisher exact tests with Benjamini–Hochberg correction: supported and high-confidence joint recovery in non-tetrapod osteichthyans versus tetrapods, and *NTF4* recovery in ray-finned fishes or non-mammalian tetrapods versus mammals. Because sampling was purposive, species are phylogenetically non-independent, and confidence depends partly on assembly and annotation quality, these tests describe sample-level evidence categories rather than clade-wide biological retention (Supplementary Table S5).

### 2.3. Phylogenetic Validation of Gene Families

Neurotrophin and Trk receptor protein sequences were aligned with MUSCLE v3.8.1551 [41] and trimmed with trimAl v1.5 using the automated1 heuristic [42]. An initial ModelFinder search on the neurotrophin alignment selected LG + F + R10. Final trimmed neurotrophin and Trk overview trees were inferred with IQ-TREE 2 v2.0.7 [43] under this predefined model, with 1000 SH-aLRT replicates and 1000 ultrafast bootstrap replicates. Trees were unrooted; display roots were placed outside the focal split and were not used for ancestral or duplication-order inference. The neurotrophin tree included *NGF*, *BDNF*, *NTF3*, and *NTF4*, whereas the Trk receptor tree included *NTRK1*, *NTRK2*, and *NTRK3*. *NGFR* was excluded from the Trk-family tree because p75NTR lies outside that receptor family. It was evaluated separately in a 90-sequence TNFR-superfamily context tree containing 65 focal *NGFR*-labeled records and 25 human TNFRSF references. These sequences were aligned with MAFFT v7.490 [44], and an unrooted tree was inferred under the predefined LG + F + R6 model with the same support scheme. Because 24 sequences failed the IQ-TREE composition chi-square test, this tree was used as family-context support rather than complete orthology reconciliation (Supplementary Figure S1; Supplementary Table S6).

### 2.4. Locus, Domain, and Non-Tetrapod Candidate Adjudication

Representative non-tetrapod *NGF* and *NGFR* candidates were evaluated using reciprocal classification, profile-HMM screening, local synteny evidence, and domain architecture. NGF-like amino acid segments derived from genomes were classified by reciprocal BLASTP v2.14.1+ against human neurotrophin-family reference proteins, including NGF, BDNF, NTF3, and NTF4 (E-value threshold 1 × 10^−3^; up to 8 targets). Initial tBLASTn hits with E ≤ 10^−8^, bit score ≥ 45, at least 4 cysteines, and length ≥ 40 amino acids were retained under permissive, project-specific screening criteria. These criteria were designed for short cysteine-rich neurotrophin candidates, with final assignments based on downstream evidence integration. Gene- and family-specific HMMs were built from the project alignments, including retained candidates, with HMMER v3.3.2 hmmbuild and searched with hmmsearch defaults [45]. HMM scores ranked family similarity within the downstream evidence framework. Human receptor feature boundaries were taken from UniProt records P04629 (NTRK1), Q16620 (NTRK2), Q16288 (NTRK3), and P08138 (NGFR). Candidate architecture was assessed from alignment coverage of the corresponding annotated extracellular, transmembrane, kinase, cysteine-rich, and death-domain regions. Local neighborhoods were extracted from GFF annotations within 2 Mb on either side of the focal locus and summarized using up to 10 upstream and 10 downstream genes. A neighboring marker was counted when candidate and reference windows shared a normalized non-LOC gene symbol or an informative normalized annotation description. Synteny assessment also considered the marker side relative to the focal gene and gene-order consistency under same-orientation or inverted-orientation models; conserved-neighbor counts remained descriptive and are reported in Supplementary Table S3. High-confidence and synteny-supported calls required concordance among reciprocal or family classification, expected sequence or domain features, and available locus evidence. Sequence-only candidates were retained when sequence evidence was plausible, but locus continuity or a complete gene model was unavailable. NTRK candidates were additionally evaluated for extracellular recognition regions, a transmembrane segment, and kinase-domain coverage.

### 2.5. Codon Alignments and dN/dS Models

Codon alignments were generated separately for each gene by translating the CDS, aligning the proteins with MAFFT v7.490 in auto mode [44], and projecting the alignment back to codons with PAL2NAL v14. Codon columns containing gaps spanning complete codons in more than 50% of sequences, or any gap spanning part of a codon, were removed. For analyses of receptor domains, sequences with > 50% gaps in the relevant partition were also excluded, and partitions shorter than 30 codons were not estimated. A separate unrooted nucleotide tree was inferred for each retained partition with IQ-TREE 2 under GTR + F + R4 using the fast search option. One-ratio codeml analyses in PAML v4.9j [46] used runmode = 0, seqtype = 1, CodonFreq = 2, model = 0, NSsites = 0, fix_kappa = 0, fix_omega = 0, initial kappa = 2, omega = 0.2, cleandata = 0, and method = 1. *NGF* was partitioned into the filtered full coding region, pro-domain (human prepro-NGF residues 19–121), and mature NGF (residues 122 to the C terminus). Receptor genes were partitioned into mutually exclusive domain classes based on human UniProt coordinates. Analyses were gene-specific and were not restricted to species with joint recovery of all three focal genes. Partition-specific ω values were estimated independently and interpreted descriptively, without a formal test of equal constraint. Original codon sets contained one representative transcript per retained accession-bearing gene record after sequence quality control; multiple records from the same sampled taxon remained separate in sequence-level analyses but contributed only one species-level recovery vote. High-confidence-only one-ratio re-fits were used as a sensitivity analysis (Supplementary Tables S4 and S7).

### 2.6. Conservation and Structural Mapping

Amino acid conservation was calculated at each position aligned to the human reference as C = 1 − H_20_, where H_20_ = −Σᵢ pᵢ log_20_(pᵢ) and pᵢ is the frequency of amino acid i among non-gap, non-X residues. Scores range from 0 to 1, with 1 indicating complete conservation. Gaps and X states were excluded from both residue frequencies and the denominator at each site. Only columns containing a human reference residue were reported; no additional post hoc coverage cutoff was applied, and the non-gap sequence count was retained for each position. All retained sequences contributed equally; no clade weighting was applied. Mature NGF conservation was mapped using PDB entries 2IFG, 1WWW, and 1SG1 and their associated publications [5,15,16]. Contacts were defined by a 4.5 Å heavy-atom threshold. Among positions with resolved coordinates, mutually exclusive classes were assigned in the order cysteine scaffold, dimer interface, TrkA contact, p75NTR contact, and other coordinate-resolved mature residues. A total of 12 mature-NGF positions without sufficient structural coordinates were excluded. Conservation distributions were compared with the other coordinate-resolved mature residues using two-sided asymptotic Mann–Whitney U tests with tie correction in SciPy 1.15.3, followed by Benjamini–Hochberg correction [47]. These exploratory residue-level tests do not model structural or phylogenetic dependence.

### 2.7. Receptor Domain Constraint Analysis

Mean amino acid conservation was calculated for mutually exclusive extracellular non-module, recognition-module, transmembrane, cytoplasmic non-domain, and kinase/death-domain receptor partitions. Recognition modules corresponded to Trk ligand-recognition domains and NGFR CRD1–4; kinase/death corresponded to Trk kinase domains or the NGFR death-domain region. dN/dS was estimated with codeml one-ratio models for all evaluable partitions. Transmembrane partitions were not estimated because they contained fewer than 30 codons; NE denotes not estimated. NTRK2 and NTRK3 kinase domains were included as Trk-family context, whereas *NGF*, *NTRK1*, and *NGFR* were the three focal genes. Filtering was performed separately for each alignment and partition.

### 2.8. Exploratory Branch and Site Analyses

Exploratory branch and site analyses were used to test for localized shifts in selective constraint. Two-ratio branch models in PAML [46] compared 9 predefined foreground sets with background branches for *NGF*, *NTRK1*, and *NGFR*, yielding 27 comparisons. The 9 foreground definitions are listed in Supplementary Table S8. *p* values from likelihood-ratio tests were adjusted across all comparisons using the Benjamini–Hochberg procedure. These models test shifts in mean constraint and do not by themselves establish positive selection. Mature NGF was evaluated for episodic selection with MEME in HyPhy [48]. Candidate sites were interpreted after Benjamini–Hochberg correction [47] and were considered supplementary to the primary recovery and structural constraint analyses.

### 2.9. Figure Generation

Figures were generated in Python 3.10 using matplotlib 3.8.4 and related scientific plotting libraries. Molecular structure panels were rendered in PyMOL 2.5.0 and assembled into the final figure layout.

## 3. Results

### 3.1. Joint Recovery Varies Across Chordate Groups

Across the 94 sampled chordates, the 16 non-gnathostome species were retained as evolutionary boundary taxa and were not included in focal-gene joint-recovery proportions. Among the 78 sampled gnathostomes, 52 species showed high-confidence joint recovery of *NGF*, *NTRK1*, and *NGFR*; eight showed supported provisional joint recovery; four had component-level evidence consistent with possible co-recovery; and 14 remained incomplete (Figure 1; Supplementary Tables S1, S2 and S5). In chondrichthyans, provisional *NGF* records were recovered in five of six species, and *NGFR* records in all six, but only *Chiloscyllium punctatum* and *Carcharodon carcharias* also had partial *NTRK1*-like models. These two species were therefore classified as possible co-recovery rather than supported joint recovery.

Among the 20 sampled ray-finned fishes, nine showed high-confidence joint recovery, six showed supported provisional joint recovery, one showed possible co-recovery, and four remained incomplete. Of the two sampled non-tetrapod sarcopterygians, *Protopterus annectens* showed high-confidence joint recovery, whereas *Latimeria chalumnae* showed supported provisional joint recovery because *NGF* remained a synteny-supported candidate. Overall, 17 of 22 non-tetrapod osteichthyans and 43 of 50 tetrapods met the supported joint-recovery definition (77.3% versus 86.0%; two-sided Fisher exact *p* = 0.493). This sample-level contrast was not significant and was not interpreted as a clade-wide prevalence estimate (Supplementary Table S5).

High-confidence joint recovery predominated among the sampled tetrapods. Seventeen of 20 mammals and 25 of 30 non-mammalian tetrapods had high-confidence *NGF*, *NTRK1*, and *NGFR* assignments. High-confidence joint recovery was more frequent in tetrapods than in non-tetrapod osteichthyans (42/50 versus 10/22; Benjamini–Hochberg-adjusted *p* = 0.0058). Within tetrapods, one mammal had supported provisional recovery, one non-mammalian species had possible co-recovery, and six species remained incomplete. Species-level evidence is provided in Supplementary Table S1, and clade-level counts are summarized in Supplementary Tables S2 and S5.

*NTF4* showed a mixed non-recovery pattern. It was recovered in 9 of 20 ray-finned fishes, 15 of 30 non-mammalian tetrapods, and 18 of 20 mammals. Recovery was less frequent in ray-finned fishes and non-mammalian tetrapods than in mammals (Benjamini–Hochberg-adjusted *p* = 0.0076 for both comparisons; Supplementary Table S5). Of the 15 non-recovered non-mammalian-tetrapod records, 12 were birds, whereas ray-finned-fish non-recoveries occurred across several distant lineages. The inventory cannot distinguish gene loss from sequence divergence or heterogeneous assembly and annotation.

### 3.2. Gene Family Phylogenies Separate NGF and NTRK1 from Their Paralogs

In the unrooted neurotrophin overview tree, *NGF* was separated from *BDNF*, *NTF3*, and *NTF4* by an exclusive edge (Figure 2A). The 71 curated *NGF* sequences had 100/100 SH-aLRT/UFBoot support; *BDNF* and *NTF3* had 98.7/100 and 95.2/98 support, respectively. The 47 displayed *NTF4* records formed an exclusive split with 95.6/100 support. An additional *Danio rerio* record was retained in the full source tree. Because the tree is unrooted, these splits support family separation but do not establish duplication order or the origin branch of *NGF*.

The Trk receptor tree separated *NTRK1* from *NTRK2* and *NTRK3* (Figure 2B). The 64 *NTRK1* sequences formed a supported exclusive split, and *NTRK2* and *NTRK3* provided paralogous context. The four cyclostome Trk-family sequences were retained in the full source tree but excluded from named *NTRK2* and focal-paralog counts. Partial kinase-dominated *NTRK1*-like models were not upgraded solely from tree proximity. In the separate *NGFR*/*TNFRSF* context tree, all 65 focal *NGFR*-labeled sequences were nearest to human *NGFR*/*TNFRSF*16 rather than the other human *TNFRSF* references in a patristic-distance screen (Supplementary Figure S1; Supplementary Table S6). Composition heterogeneity required final *NGFR* calls to retain locus and architecture evidence.

### 3.3. Locus and Domain Evidence Refine Non-Tetrapod Candidate Assignments

We next examined boundary cases whose classification could alter a species-level recovery category or distinguish a focal assignment from an ancestral or paralogous hit. Figure 3A,B show representative component-level adjudications rather than same-species reconstruction of a functional pathway.

The *Latimeria chalumnae* and *Acipenser ruthenus NGF* candidates were reciprocally classified as *NGF* and retained conserved *NGF*-neighborhood markers (Figure 3A). The *Latimeria chalumnae* locus contained 10 conserved neighboring markers, whereas the *Acipenser ruthenus* locus contained seven. Both were retained as provisional *NGF* assignments. Reanalysis also identified a provisional *Scyliorhinus canicula NGF* record with reciprocal *NGF* support and a CD9/KCNA-associated neighborhood, consistent with catshark neurotrophin context [49]. In contrast, the cyclostome *NGF*-like signal was reciprocally classified as *NTF3*-like and lacked an *NGF* neighborhood.

The *Lepisosteus oculatus NGFR* candidate contained four cysteine-rich domains, a transmembrane region, and a death domain, together with 11 conserved locus markers (Figure 3B). This evidence supported a provisional *NGFR* assignment. The *Scyliorhinus canicula NGFR* record had complete p75NTR-compatible architecture and reciprocal *NGFR* support and was assigned with high confidence, consistent with independent characterization of catshark p75NTR [50]. The *Callorhinchus milii* receptor-like signal showed only partial TNFR-like architecture and remained a sequence-level candidate. Earlier receptor- or domain-like signals with partial CRD-like coverage and no canonical locus support were not assigned as *NGFR*.

At the species level, *Lepisosteus oculatus* and *Latimeria chalumnae* each showed supported provisional joint recovery. *Protopterus annectens* showed high-confidence joint recovery of all three focal genes. The partial *NTRK1*-like records in *Chiloscyllium punctatum* and *Carcharodon carcharias* set a sequence-only ceiling, so these chondrichthyans remained possible co-recovery cases. The *Acipenser ruthenus NGF* candidate provided component-level evidence but did not support joint recovery. Component-level evidence in a lineage was not used to infer that all three loci were present in its ancestor.

### 3.4. Mature NGF Shows Region-Specific Constraint

Mean amino acid conservation was 0.58 across the NGF pro-domain and 0.84 across mature NGF (Figure 4A). The independently fitted codon models showed the same numerical ordering: ω was 0.175 for the full coding region, 0.352 for the pro-domain, and 0.067 for mature NGF (Figure 4B; Supplementary Table S9). The original *NGF* set comprised 69 high-confidence assignments and two synteny-supported candidates with complete coding sequences; high-confidence-only re-fitting retained the low mature-NGF estimate and the same numerical ordering (Supplementary Table S7).

Within mature NGF, the six scaffold cysteines were completely conserved, and much of the dimer interface also showed high conservation (Figure 4C,D). More variable positions occurred in several peripheral regions of the mature domain. Residues contacting TrkA or p75NTR were conserved, but their conservation occurred against a background of generally high conservation across the mature domain.

Under the mutually exclusive hierarchical classification used for statistical comparison, the six scaffold cysteines each had a conservation score of 1.000 and differed from the background group of 42 other mature residues with resolved coordinates (Benjamini–Hochberg-adjusted *p* = 0.021; Figure 4E). The dimer-interface, TrkA-contact, and p75NTR-contact classes did not differ significantly from the background after correction (Supplementary Table S10). This exploratory Mann–Whitney analysis is limited by the small cysteine sample, ties, and structural and evolutionary dependence among residue positions. A total of 12 positions lacking sufficient coordinate coverage were excluded rather than assigned to the background.

### 3.5. Intracellular Receptor Regions Show Lower ω Estimates

Amino acid conservation varied among receptor partitions (Figure 5B). The Trk kinase domains showed mean conservation values of 0.912 for NTRK1, 0.959 for NTRK2, and 0.955 for NTRK3. In NGFR, the transmembrane region had the highest mean conservation (0.931), whereas the extracellular non-module region had the lowest value (0.549). Recognition modules and extracellular non-module regions remained conserved but were generally more variable than the Trk kinase domains.

The lowest evaluable ω estimates occurred in intracellular receptor regions (Figure 5C). The NTRK1, NTRK2, and NTRK3 kinase domains had ω values of 0.026, 0.014, and 0.014, respectively. Within NGFR, the cytoplasmic non-domain region had ω = 0.051, and the death domain had ω = 0.063. Transmembrane partitions were not estimated because they contained fewer than 30 codons; NE denotes not estimated and does not imply absence of selection. High-confidence-only refits preserved low estimates in the receptor signaling partitions (Supplementary Tables S7 and S11).

Across the eight extracellular receptor partitions, the median ω was 0.164 (Figure 5D). The lower estimates in the Trk kinase domains and NGFR intracellular regions were descriptive because the partitions were fitted independently. NTRK2 and NTRK3 provide Trk-family context rather than canonical NGF-axis components. Receptor conservation and codon-model estimates are provided in Supplementary Tables S11 and S12.

### 3.6. Exploratory Analyses Show Branch-Level Variation in Constraint

Branch analyses were used as exploratory tests for localized shifts within an overall model of purifying selection. In two-ratio branch models for *NGF*, *NTRK1*, and *NGFR* [46], all tested foreground mean ω estimates remained below 1. Of the 27 comparisons, 14 remained significant after correction, indicating heterogeneity in constraint strength rather than evidence that foreground means exceeded neutrality.

MEME scans of mature NGF [48] identified five sites with unadjusted *p* ≤ 0.1, but no site remained significant after Benjamini–Hochberg correction. No statistically supported episodic diversifying-selection site was therefore detected under the present alignment and model. Exploratory branch and MEME results are provided in Supplementary Table S8.

## 4. Discussion

Previous studies reconstructed the evolution of neurotrophin and Trk receptor families and linked their expansion and diversification to early vertebrate genome duplications [21,22,23]. Those studies established that neurotrophin signaling predates vertebrates, and thus the emergence of *NGF* and *NTRK1* as distinct paralogs in gnathostomes [22,26,27,28]. The present analysis extends this work by evaluating *NGF*, *NTRK1*, and *NGFR* across 94 chordates using graded orthology evidence and paralog context, a confidence-based framework that distinguishes high-confidence assignments from provisional and sequence-only cases. The non-gnathostome species provide the evolutionary boundary for interpreting these results. Among sampled gnathostomes, the main novel contribution was not simple recovery but assignment confidence. Joint recovery was common in both non-tetrapod osteichthyans and tetrapods, whereas high-confidence assignments were proportionally more frequent in tetrapods. Separating high-confidence from provisional and sequence-only cases revealed differences that would be obscured by a binary recovery summary.

Previous analyses of selected invertebrates considered neurotrophin, Trk, and p75NTR homologs together [29,30], whereas teleost studies examined ligand-receptor retention after genome duplication [51]. Our non-tetrapod candidate assessments illustrate the practical effect of graded evidence on orthology calls. The shark reanalysis also showed that a single-best-hit screen can favor an *NTF3* locus and mask a second *NGF*-like locus. Provisional *NGF* assignments were recovered in *Latimeria chalumnae*, *Acipenser ruthenus*, and five chondrichthyans. A total of four chondrichthyan records had locus support, whereas the *Callorhinchus milii* record was sequence-supported and was not mapped to the current reference assembly. A p75NTR-compatible *NGFR* candidate was retained in *Lepisosteus oculatus*, whereas partial *NTRK1*-like signals were not upgraded beyond sequence-only status. This distinction is compatible with previous reports of chondrichthyan neurotrophin and p75NTR conservation [49,50], though divergence, assembly gaps, or incomplete annotation remain alternative explanations.

Evolutionary constraint was unevenly distributed within NGF. Earlier studies established the overall conservation of vertebrate NGF and examined evolutionary patterns in proneurotrophins [13,25]. The present analysis adds direct comparisons between predefined structural classes in mature NGF and other mature residues with resolved coordinates. Independent fits yielded a lower ω estimate for mature NGF than for the pro-domain (0.067 versus 0.352; Figure 4B). Within the mature domain, the invariant cysteines form the cystine-knot scaffold; substitutions at these positions would be expected to disrupt disulfide pairing, folding, or secretion [15,16,36]. The dimer interface also showed high mean conservation, but it was not significant after correction. TrkA- and p75NTR-contacting residues likewise did not differ from the background. High-confidence-only re-fitting retained the same regional ordering.

Constraint also varied among receptor regions. Including NGFR allowed regional constraint in p75NTR to be compared with that in Trk receptors, which belong to a different receptor family. Trk kinase domains retain catalytic and phosphorylation-dependent signaling machinery, whereas the p75NTR death domain supports conserved adaptor interactions [6,17,18]. Their low ω estimates are consistent with strong purifying selection, whereas extracellular regions were comparatively more permissive without being unconstrained. Across the branch and site analyses, all foreground mean ω estimates remained below 1, and no mature-NGF site remained significant after correction [46,48]. The principal evolutionary pattern detected in these analyses was regional variation in the strength of purifying constraint.

NGF-TrkA-p75NTR signaling contributes to pain, repair, osteoarthritic remodeling, tendon fibrosis, and cancer treatment response [4,8,9,10,11,12]. The invariant cysteine scaffold and low-ω intracellular receptor regions highlight priorities for targeted functional testing, whereas the more permissive pro-domain and peripheral mature-NGF positions may accommodate lineage- or context-dependent variation. Testing these possibilities will require measurements of folding, secretion, binding, receptor activation, and tissue-specific expression.

Recovery calls are influenced by assembly and annotation quality, and because the species panel prioritized phylogenetic breadth over proportional representation, the reported proportions describe the sampled dataset rather than clade-wide prevalence. A complete historical conflict matrix for every external discovery database could not be reconstructed because all intermediate discovery outputs were not retained. Functional integration cannot be inferred from joint genomic recovery alone and requires expression and biochemical evidence. Partition-specific models were fitted independently, structural tests depend on the selected contact threshold and class hierarchy, and residue-level tests do not model structural or evolutionary dependence. Because sequences were equally weighted, conservation scores also reflect the taxonomic composition and retained record multiplicity of the sequence sets. Future work should combine improved assemblies and long-read transcript validation with matched-tissue expression studies. Genomic PCR or targeted RT-PCR could test partial loci, whereas species-matched ligand-receptor assays and structure-guided mutagenesis could examine folding, secretion, dimerization, receptor activation, and downstream signaling.

## 5. Conclusions

*NGF*, *NTRK1*, and *NGFR* were jointly recovered in most of the sampled non-tetrapod osteichthyans and tetrapods, but records meeting the study’s high-confidence criteria were more frequent among the sampled tetrapods. Mature NGF had a numerically lower estimated ω than the pro-domain; the cysteine scaffold was invariant, whereas conservation scores for the dimer-interface and receptor-contact classes did not differ significantly from those of other coordinate-resolved mature-domain residues after correction. Trk kinase and p75NTR intracellular regions also had low ω estimates. These results distinguish high-confidence and supported provisional joint recovery of *NGF*, *NTRK1* and *NGFR* from possible co-recovery based on sequence-only evidence and highlight regions of particularly strong evolutionary constraint in the three focal genes. Targeted locus validation, species-matched expression and receptor assays, and structure-guided tests of constrained residues provide the next steps toward functional interpretation.

## Figures and Tables

**Figure 1:**
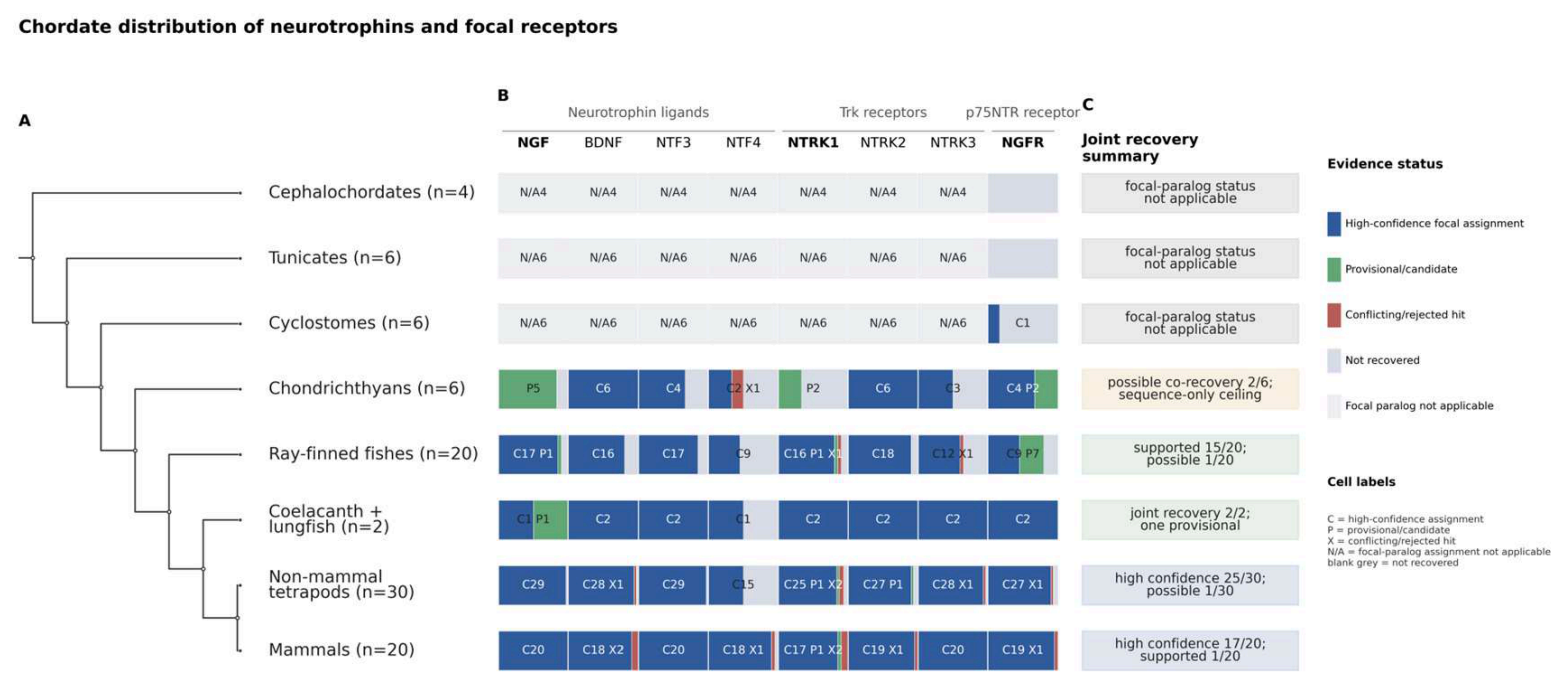
Evidence-graded distribution of neurotrophins and focal receptors: (**A**) Collapsed taxonomic framework for the 94 surveyed chordates. (**B**) Species-level evidence composition for *NGF*, *BDNF*, *NTF3*, *NTF4*, *NTRK1*, *NTRK2*, *NTRK3*, and *NGFR*. *NGF*, *NTRK1*, and *NGFR* define the focal set; the other genes provide paralog context. Cell labels report nonzero counts: C, high-confidence focal assignment; P, provisional or sequence-only candidate; X, conflicting/rejected hit; N/A, focal-paralog assignment not applicable. Blank gray cells indicate no recovered candidate under the present criteria and do not establish biological absence. Named neurotrophin and Trk paralogs in non-gnathostome boundary taxa are N/A; family-level homologs are reported in Supplementary Table S3. (**C**) Qualitative clade-level summary of joint recovery of *NGF*, *NTRK1*, and *NGFR*. Supported joint recovery requires all three genes in one species with no sequence-only component; sequence-only evidence defines possible co-recovery. Sequence-only candidates are included within P in panel (**B**) but are separated from supported provisional recovery in panel (**C**).

**Figure 2:**
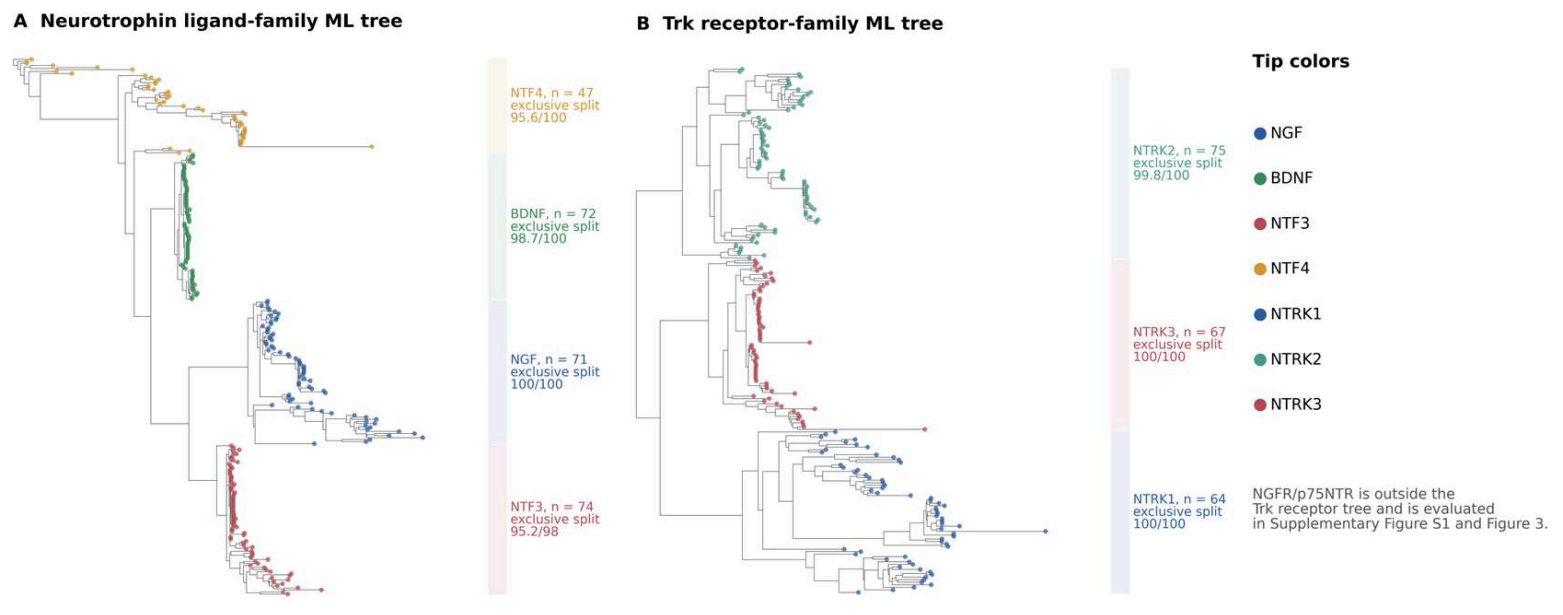
Family-level phylogenetic validation: (**A**) Unrooted maximum-likelihood overview of *NGF*, *BDNF*, *NTF3*, and *NTF4*. (**B**) Unrooted overview of *NTRK1, NTRK2*, and *NTRK3*. Tip colors denote annotated paralog identity; support values are SH-aLRT/UFBoot percentages for the displayed exclusive split. Counts beside a split exclude boundary or display-omitted records noted in the text. Display rooting is graphical and carries no ancestral interpretation. Full labeled trees and complete support values are supplied in Supplementary Data S1. *NGFR*/p75NTR lies outside the Trk family and is evaluated separately by TNFRSF family context, locus, and architecture evidence (Supplementary Figure S1; Figure 3).

**Figure 3:**
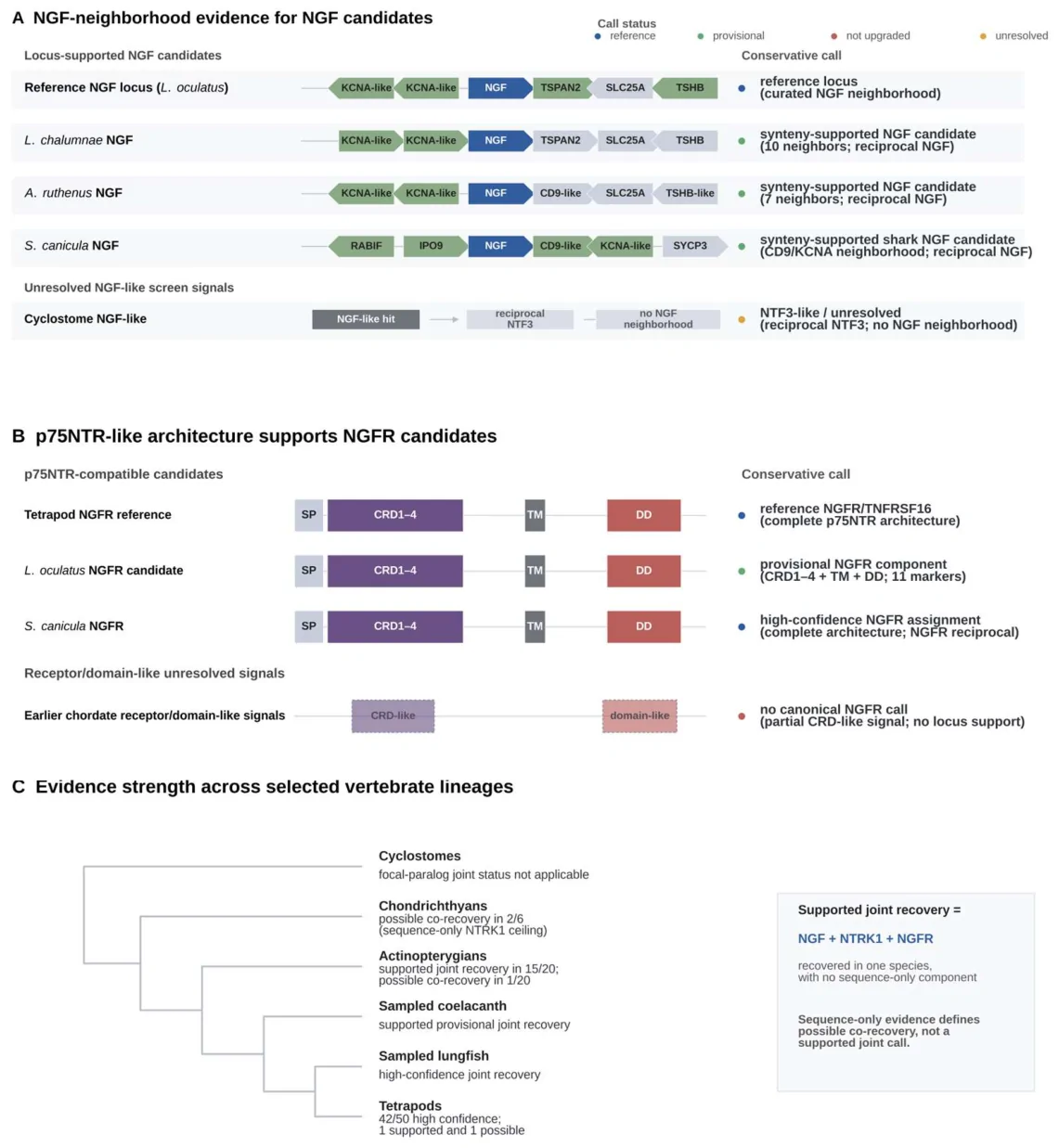
Locus and domain evidence for selected non-tetrapod candidates: (**A**) GFF-derived, non-scale gene-order schematics centered on *NGF* show representative conserved neighbors; unresolved rows summarize screen outcomes. Conserved-neighbor counts refer to the full screened neighborhood. (**B**) Schematic p75NTR-compatible architectures used in *NGFR* adjudication. CRD1–4 denotes TNFR cysteine-rich domains; TM, transmembrane region; DD, death domain. Dashed pale blocks denote partial domain-like signals. Domain diagrams are not drawn to scale. (**C**) Evidence strength across selected vertebrate lineages. The 2 chondrichthyans are classified as possible co-recovery; supported recovery in other groups requires all three genes with no sequence-only component. Panels (**A**,**B**) are representative component-level adjudications and do not establish same-species pathway function. Blue arrows indicate focal *NGF*, muted green arrows indicate representative conserved neighboring genes, and gray arrows indicate contextual genes.

**Figure 4:**
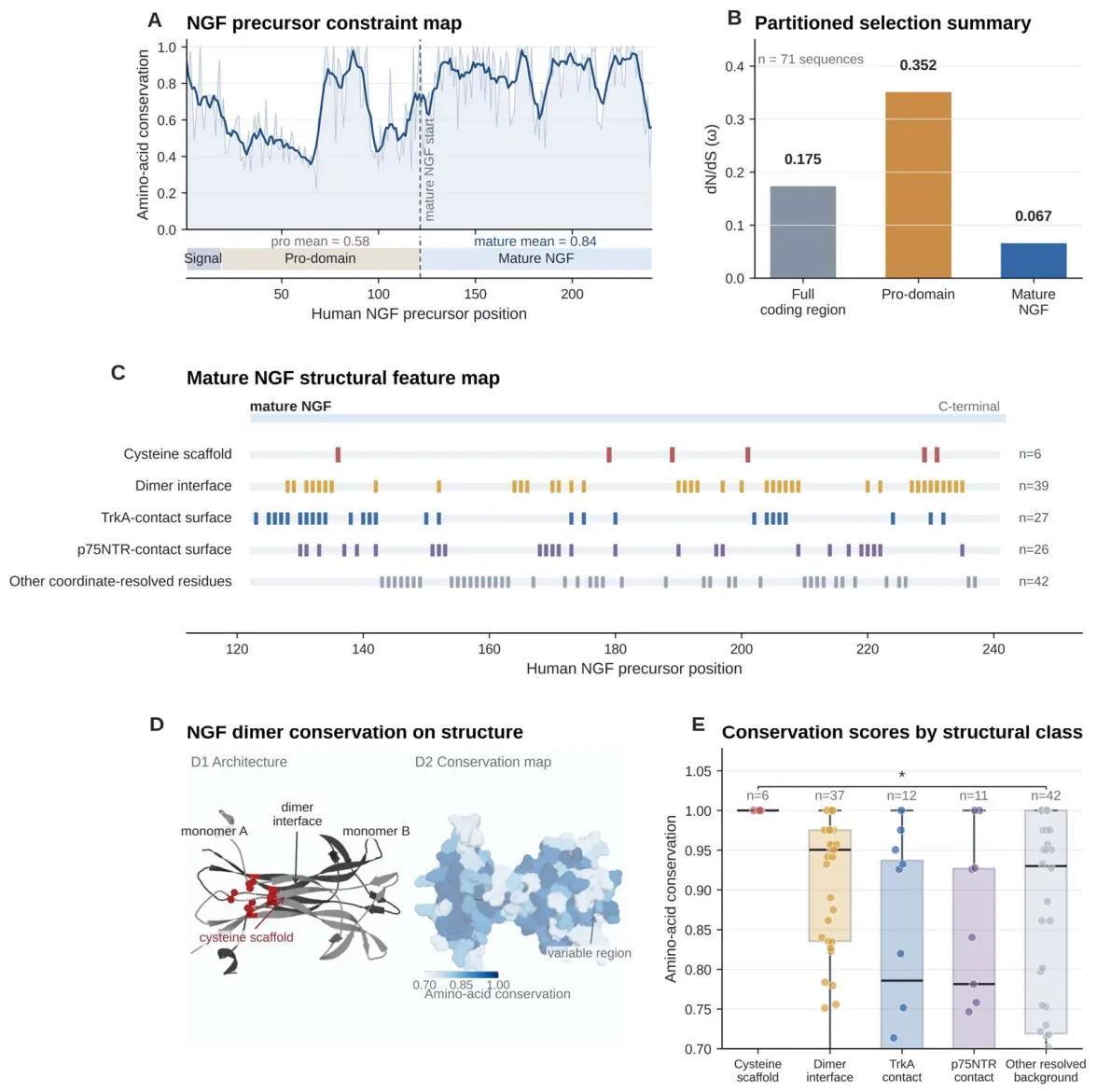
Regional and structural conservation of NGF: (**A**) Amino acid conservation across the human NGF precursor sequence, with the signal peptide, pro-domain, and mature NGF indicated below the profile. Scores range from 0 to 1, with higher values indicating greater conservation. (**B**) Independent ω point estimates for the full coding region, pro-domain, and mature NGF. (**C**) Overlapping structural-feature tracks across mature NGF in human precursor coordinates. Contacts used a 4.5 Å heavy-atom threshold; solvent accessibility was not used to define the background. (**D**) NGF homodimer architecture and conservation mapped using PDB 2IFG; receptor contacts were cross-checked against PDB 1WWW and 1SG1 [5,15,16]. (**E**) Conservation distributions for mutually exclusive structural classes. Points represent residues; boxes show the interquartile range, horizontal lines show medians, and whiskers extend to the most extreme values within 1.5 × IQR. Other coordinate-resolved mature residues were the background. A total of 12 positions without sufficient coordinate coverage were excluded. * Benjamini–Hochberg-adjusted *p* < 0.05.

**Figure 5:**
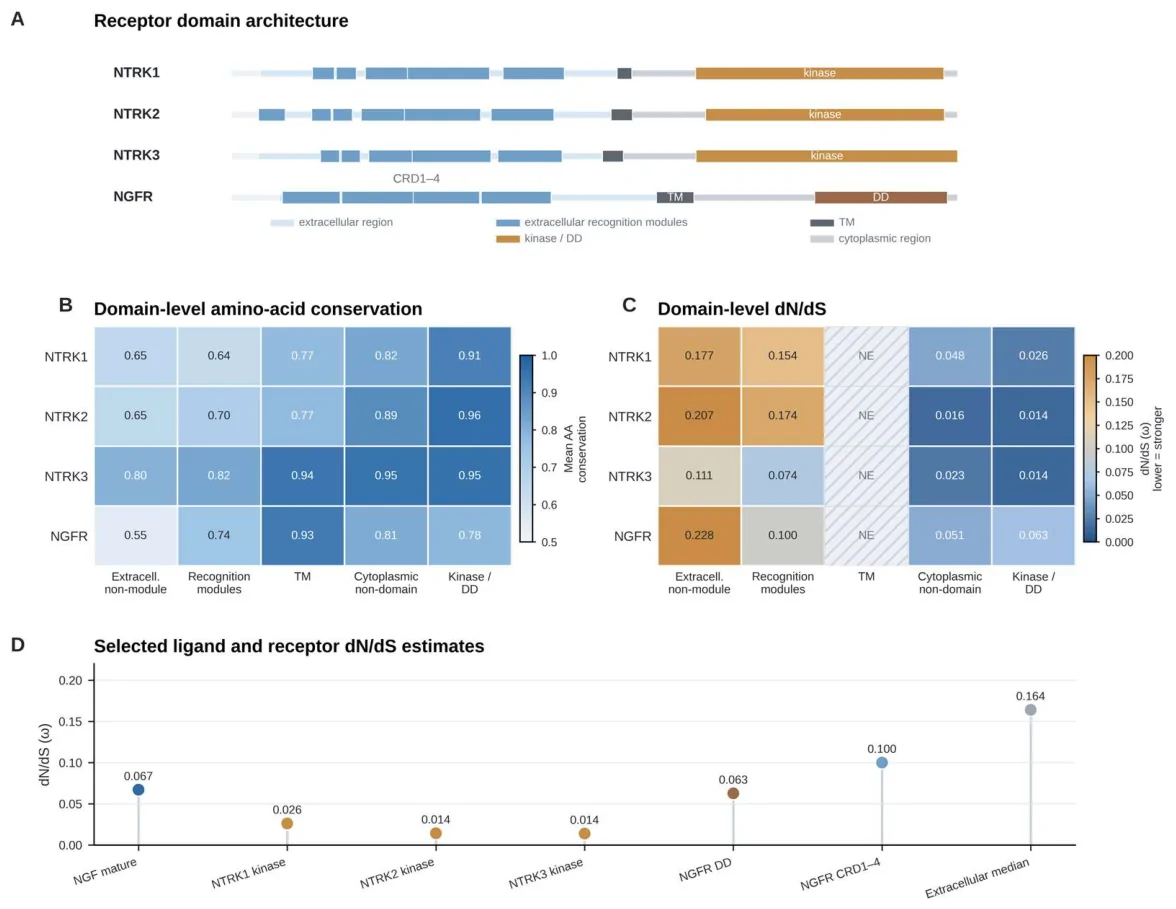
Conservation and evolutionary constraint across receptor partitions: (**A**) Simplified, non-scale domain schematics of human NTRK1, NTRK2, NTRK3, and NGFR. Recognition modules denote extracellular ligand-recognition domains in Trk receptors and CRD1–4 in NGFR; DD denotes death domain. (**B**) Mean amino acid conservation for mutually exclusive receptor partitions. (**C**) One-ratio ω estimates for the same partitions; lower values indicate stronger purifying constraint. Hatched transmembrane cells are labeled NE because these partitions were too short for reliable estimation. (**D**) Descriptive estimates for selected ligand and receptor regions. NTRK2 and NTRK3 provide Trk-family context. The extracellular median is the median across extracellular-recognition and extracellular non-module partitions.

## Data Availability

The original contributions presented in this study are included in the article/Supplementary Materials. Further inquiries can be directed to the corresponding author.

## Supplementary Materials

The following supporting information can be downloaded at: https://www.mdpi.com/article/10.3390/biology15191695/s1, Figure S1. NGFR/TNFRSF family-context validation; Table S1. Species-level focal-assignment evidence matrix; Table S2. Clade-level focal-assignment and joint-recovery summary; Table S3. Boundary-lineage accession reconciliation and candidate adjudication; Table S4. Evidence-state definitions and analysis inclusion rules; Table S5. Recovery-category sensitivity and exploratory sample contrasts; Table S6. Family-level phylogenetic validation summary; Table S7. High-confidence-only codon-model sensitivity analysis; Table S8. Exploratory branch and site-level selection scans; Table S9. NGF partitioned codon-model ω estimates; Table S10. NGF structural-class conservation source data; Table S11. Receptor domain codon-model ω estimates; Table S12. Receptor domain conservation source data; Data S1. Full labeled neurotrophin and Trk family trees, associated trimmed amino-acid alignments, sequence-label mapping tables, and IQ-TREE support reports.

## Abbreviations

The following abbreviations are used in this manuscript:

*BDNF*	Brain-derived neurotrophic factor
BLASTP	Basic Local Alignment Search Tool for proteins
CDS	Coding sequence
CRD	Cysteine-rich domain
DD	Death domain
*d*N/*d*S	Ratio of nonsynonymous to synonymous substitution rates
FDR	False discovery rate
GFF	General Feature Format
HMM	Hidden Markov model
HMMER	Profile hidden Markov model search software
HyPhy	Hypothesis Testing Using Phylogenies
MAFFT	Multiple Alignment using Fast Fourier Transform
MEME	Mixed effects model of evolution
MUSCLE	Multiple Sequence Comparison by Log-Expectation
N/A	Not applicable for focal-paralog assignment
NE	Not estimated
NCBI	National Center for Biotechnology Information
*NGF*	Nerve growth factor
*NGFR*	Nerve growth factor receptor
*NTF3*	Neurotrophin 3
*NTF4*	Neurotrophin 4
*NTRK1*	Neurotrophic receptor tyrosine kinase 1
*NTRK2*	Neurotrophic receptor tyrosine kinase 2
*NTRK3*	Neurotrophic receptor tyrosine kinase 3
p75NTR	p75 neurotrophin receptor
PAL2NAL	Program for converting a protein alignment to a codon alignment
PAML	Phylogenetic Analysis by Maximum Likelihood
PDB	Protein Data Bank
SH-aLRT	Shimodaira–Hasegawa approximate likelihood-ratio test
SP	Signal peptide
tBLASTn	Protein-query search against a translated nucleotide database
TM	Transmembrane region
TNFR	Tumor necrosis factor receptor
*TNFRSF16*	Tumor necrosis factor receptor superfamily member 16
Trk	Tropomyosin receptor kinase
TrkA	Tropomyosin receptor kinase A
UFBoot	Ultrafast bootstrap

## References

[1] Levi-Montalcini R. (1987). The nerve growth factor 35 years later. Science.

[2] Huang E.J., Reichardt L.F. (2001). Neurotrophins: Roles in neuronal development and function. Annu. Rev. Neurosci..

[3] Kaplan D.R., Hempstead B.L., Martin-Zanca D., Chao M.V., Parada L.F. (1991). The trk proto-oncogene product: A signal transducing receptor for nerve growth factor. Science.

[4] Denk F., Bennett D.L., McMahon S.B. (2017). Nerve Growth Factor and Pain Mechanisms. Annu. Rev. Neurosci..

[5] Wiesmann C., Ultsch M.H., Bass S.H., de Vos A.M. (1999). Crystal structure of nerve growth factor in complex with the ligand-binding domain of the TrkA receptor. Nature.

[6] Chao M.V. (2003). Neurotrophins and their receptors: A convergence point for many signaling pathways. Nat. Rev. Neurosci..

[7] Lu B., Pang P.T., Woo N.H. (2005). The yin and yang of neurotrophin action. Nat. Rev. Neurosci..

[8] Lee S., Hwang C., Marini S., Tower R.J., Qin Q., Negri S., Pagani C.A., Sun Y., Stepien D.M., Sorkin M. (2021). NGF-TrkA signaling dictates neural ingrowth and aberrant osteochondral differentiation after soft tissue trauma. Nat. Commun..

[9] Xu J., Li Z., Tower R.J., Negri S., Wang Y., Meyers C.A., Sono T., Qin Q., Lu A., Xing X. (2022). NGF-p75 signaling coordinates skeletal cell migration during bone repair. Sci. Adv..

[10] Zhao L., Lai Y., Jiao H., Huang J. (2024). Nerve growth factor receptor limits inflammation to promote remodeling and repair of osteoarthritic joints. Nat. Commun..

[11] Li Y., Pang S., Zhang X., Lu M., Zhao J., Wu R., Hu J., Jiang J., Chen H., Ling Z. (2026). Nociceptive sensory neuron-derived NGF orchestrates a fibrotic mesenchymal stromal cell neurogenic niche to drive tendon pathological fibrosis. Nat. Commun..

[12] Xu M., Zheng Y., Zhao L., Wang S., Chen J., Fu Z., Ma A., Liang B., Huang Y., Xu H. (2026). Activation of Nerve Growth Factor signaling limits the response to lenvatinib in hepatocellular carcinoma. Signal Transduct. Target. Ther..

[13] Covaceuszach S., Peche L.Y., Konarev P.V., Lamba D. (2021). A combined evolutionary and structural approach to disclose the primary structural determinants essential for proneurotrophins biological functions. Comput. Struct. Biotechnol. J..

[14] Dasgupta S., Pandya M.A., Zanin J.P., Liu T., Sun Q., Li H., Friedman W.J. (2024). ProNGF elicits retrograde axonal degeneration of basal forebrain neurons through p75NTR and induction of amyloid precursor protein. Sci. Signal..

[15] He X.L., Garcia K.C. (2004). Structure of nerve growth factor complexed with the shared neurotrophin receptor p75. Science.

[16] Wehrman T., He X., Raab B., Dukipatti A., Blau H., Garcia K.C. (2007). Structural and mechanistic insights into nerve growth factor interactions with the TrkA and p75 receptors. Neuron.

[17] Conroy J.N., Coulson E.J. (2022). High-affinity TrkA and p75 neurotrophin receptor complexes: A twisted affair. J. Biol. Chem..

[18] Lin Z., Tann J.Y., Goh E.T., Kelly C., Lim K.B., Gao J.F., Ibanez C.F. (2015). Structural basis of death domain signaling in the p75 neurotrophin receptor. eLife.

[19] Hallbook F., Ibanez C.F., Persson H. (1991). Evolutionary studies of the nerve growth factor family reveal a novel member abundantly expressed in *Xenopus* ovary. Neuron.

[20] Kullander K. (1992). Function and evolution in the NGF family and its receptors. J. Neurosci. Res..

[21] Hallbook F. (1999). Evolution of the vertebrate neurotrophin and Trk receptor gene families. Curr. Opin. Neurobiol..

[22] Hallbook F., Wilson K., Thorndyke M., Olinski R.P. (2006). Formation and evolution of the chordate neurotrophin and Trk receptor genes. Brain Behav. Evol..

[23] Lanave C., Colangelo A.M., Saccone C., Alberghina L. (2007). Molecular evolution of the neurotrophin family members and their Trk receptors. Gene.

[24] Dos Santos S., Mazan S., Venkatesh B., Cohen-Tannoudji J., Querat B. (2011). Emergence and evolution of the glycoprotein hormone and neurotrophin gene families in vertebrates. BMC Evol. Biol..

[25] Sunagar K., Fry B.G., Jackson T.N.W., Casewell N.R., Undheim E.A.B., Vidal N., Ali S.A., King G.F., Vasudevan K., Vasconcelos V. (2013). Molecular evolution of vertebrate neurotrophins: Co-option of the highly conserved nerve growth factor gene into the advanced snake venom arsenal. PLoS ONE.

[26] Benito-Gutierrez E., Nake C., Llovera M., Comella J.X., Garcia-Fernandez J. (2005). The single AmphiTrk receptor highlights increased complexity of neurotrophin signalling in vertebrates and suggests an early role in developing sensory neuroepidermal cells. Development.

[27] Hallbook F., Lundin L.G., Kullander K. (1998). *Lampetra fluviatilis* neurotrophin homolog, descendant of a neurotrophin ancestor, discloses the early molecular evolution of neurotrophins in the vertebrate subphylum. J. Neurosci..

[28] Satou Y., Sasakura Y., Yamada L., Imai K.S., Satoh N., Degnan B. (2003). A genomewide survey of developmentally relevant genes in *Ciona intestinalis*. V. Genes for receptor tyrosine kinase pathway and Notch signaling pathway. Dev. Genes Evol..

[29] Wilson K.H.S. (2009). The genome sequence of the protostome *Daphnia pulex* encodes respective orthologues of a neurotrophin, a Trk and a p75NTR: Evolution of neurotrophin signaling components and related proteins in the bilateria. BMC Evol. Biol..

[30] Lauri A., Bertucci P., Arendt D. (2016). Neurotrophin, p75, and Trk signaling module in the developing nervous system of the marine annelid *Platynereis dumerilii*. BioMed Res. Int..

[31] Sonnhammer E.L.L., Koonin E.V. (2002). Orthology, paralogy and proposed classification for paralog subtypes. Trends Genet..

[32] Nakatani Y., Shingate P., Ravi V., Pillai N.E., Prasad A., McLysaght A., Venkatesh B. (2021). Reconstruction of proto-vertebrate, proto-cyclostome and proto-gnathostome genomes provides new insights into early vertebrate evolution. Nat. Commun..

[33] Marlétaz F., Timoshevskaya N., Timoshevskiy V.A., Parey E., Simakov O., Gavriouchkina D., Suzuki M., Kubokawa K., Brenner S., Smith J.J. (2024). The hagfish genome and the evolution of vertebrates. Nature.

[34] Dehal P., Boore J.L. (2005). Two rounds of whole genome duplication in the ancestral vertebrate. PLoS Biol..

[35] Venkatesh B., Lee A.P., Ravi V., Maurya A.K., Lian M.M., Swann J.B., Ohta Y., Flajnik M.F., Sutoh Y., Kasahara M. (2014). Elephant shark genome provides unique insights into gnathostome evolution. Nature.

[36] McDonald N.Q., Lapatto R., Rust J.M., Gunning J., Wlodawer A., Blundell T.L. (1991). New protein fold revealed by a 2.3-A resolution crystal structure of nerve growth factor. Nature.

[37] O’Leary N.A., Cox E., Holmes J.B., Anderson W.R., Falk R., Hem V., Tsuchiya M.T.N., Schuler G.D., Zhang X., Torcivia J. (2024). Exploring and retrieving sequence and metadata for species across the tree of life with NCBI Datasets. Sci. Data.

[38] The UniProt Consortium (2023). UniProt: The Universal Protein Knowledgebase in 2023. Nucleic Acids Res..

[39] Kuznetsov D., Tegenfeldt F., Manni M., Seppey M., Berkeley M., Kriventseva E.V., Zdobnov E.M. (2023). OrthoDB v11: Annotation of orthologs in the widest sampling of organismal diversity. Nucleic Acids Res..

[40] Herrero J., Muffato M., Beal K., Fitzgerald S., Gordon L., Pignatelli M., Vilella A.J., Searle S.M.J., Amode R., Brent S. (2016). Ensembl comparative genomics resources. Database.

[41] Edgar R.C. (2004). MUSCLE: Multiple sequence alignment with high accuracy and high throughput. Nucleic Acids Res..

[42] Capella-Gutierrez S., Silla-Martinez J.M., Gabaldon T. (2009). trimAl: A tool for automated alignment trimming in large-scale phylogenetic analyses. Bioinformatics.

[43] Minh B.Q., Schmidt H.A., Chernomor O., Schrempf D., Woodhams M.D., von Haeseler A., Lanfear R. (2020). IQ-TREE 2: New models and efficient methods for phylogenetic inference in the genomic era. Mol. Biol. Evol..

[44] Katoh K., Standley D.M. (2013). MAFFT multiple sequence alignment software version 7: Improvements in performance and usability. Mol. Biol. Evol..

[45] Eddy S.R. (2011). Accelerated profile HMM searches. PLoS Comput. Biol..

[46] Yang Z. (2007). PAML 4: Phylogenetic analysis by maximum likelihood. Mol. Biol. Evol..

[47] Benjamini Y., Hochberg Y. (1995). Controlling the false discovery rate: A practical and powerful approach to multiple testing. J. R. Stat. Soc. B.

[48] Kosakovsky Pond S.L., Poon A.F.Y., Velazquez R., Weaver S., Hepler N.L., Murrell B., Shank S.D., Magalis B.R., Bouvier D., Nekrutenko A. (2020). HyPhy 2.5: A customizable platform for evolutionary hypothesis testing using phylogenies. Mol. Biol. Evol..

[49] Chiavacci E., Bagnoli S., Cellerino A., Terzibasi Tozzini E. (2023). Distribution of brain-derived neurotrophic factor in the brain of the small-spotted catshark *Scyliorhinus canicula*, and evolution of neurotrophins in basal vertebrates. Int. J. Mol. Sci..

[50] Chiavacci E., Camera R., Costa M., Fronte B., Terzibasi Tozzini E., Cellerino A. (2025). Nerve Growth Factor Receptor (NGFR/p75NTR) of the Small-Spotted Catshark (*Scyliorhinus canicula*): Evolutionary Conservation and Brain Function. J. Comp. Neurol..

[51] Grandchamp A., Piégu B., Monget P. (2019). Genes Encoding Teleost Fish Ligands and Associated Receptors Remained in Duplicate More Frequently than the Rest of the Genome. Genome Biol. Evol..

