# Supplementary material for: Comparative Genomics of NGF, NTRK1, and NGFR Across Chordates: Joint Gene Recovery and Region-Specific Constraint
Source: Biology (Basel). 2026 Sep 23;15(19):1695. doi: 10.3390/biology15191695 (PMC13663574; doi:10.3390/biology15191695)
Supplement: Supplementary file 1 [file biology-15-01695-s001.zip › Supplementary_Figure_Legend_S1.pdf]

## Supplementary Figure Legend

**Supplementary Figure S1. NGFR/TNFRSF family-context validation.** Unrooted maximum-likelihood tree of 65 focal NGFR-labeled sequences and 25 human TNFR-superfamily references. Sequences were aligned with MAFFT v7.490, and the tree was inferred in IQ-TREE 2 under LG+F+R6 with 1,000 SH-aLRT and 1,000 ultrafast-bootstrap replicates. All focal records were nearest to human NGFR/TNFRSF16 rather than the other 24 human TNFRSF references in a patristic-distance screen. Of the 90 sequences, 24 failed the IQ-TREE composition chi-square test at $P < 0.05$; the tree is therefore used as family-context support rather than complete orthology reconciliation. Final NGFR assignments also required compatible architecture and locus evidence. The display root is graphical and has no ancestral interpretation. Human TNFRSF references are shown in gray, and selected early/boundary candidates are shown in bold.
