# Supplementary material for: Comparative Genomics of NGF, NTRK1, and NGFR Across Chordates: Joint Gene Recovery and Region-Specific Constraint
Source: Biology (Basel). 2026 Sep 23;15(19):1695. doi: 10.3390/biology15191695 (PMC13663574; doi:10.3390/biology15191695)

NGFR/TNFRSF validation tree

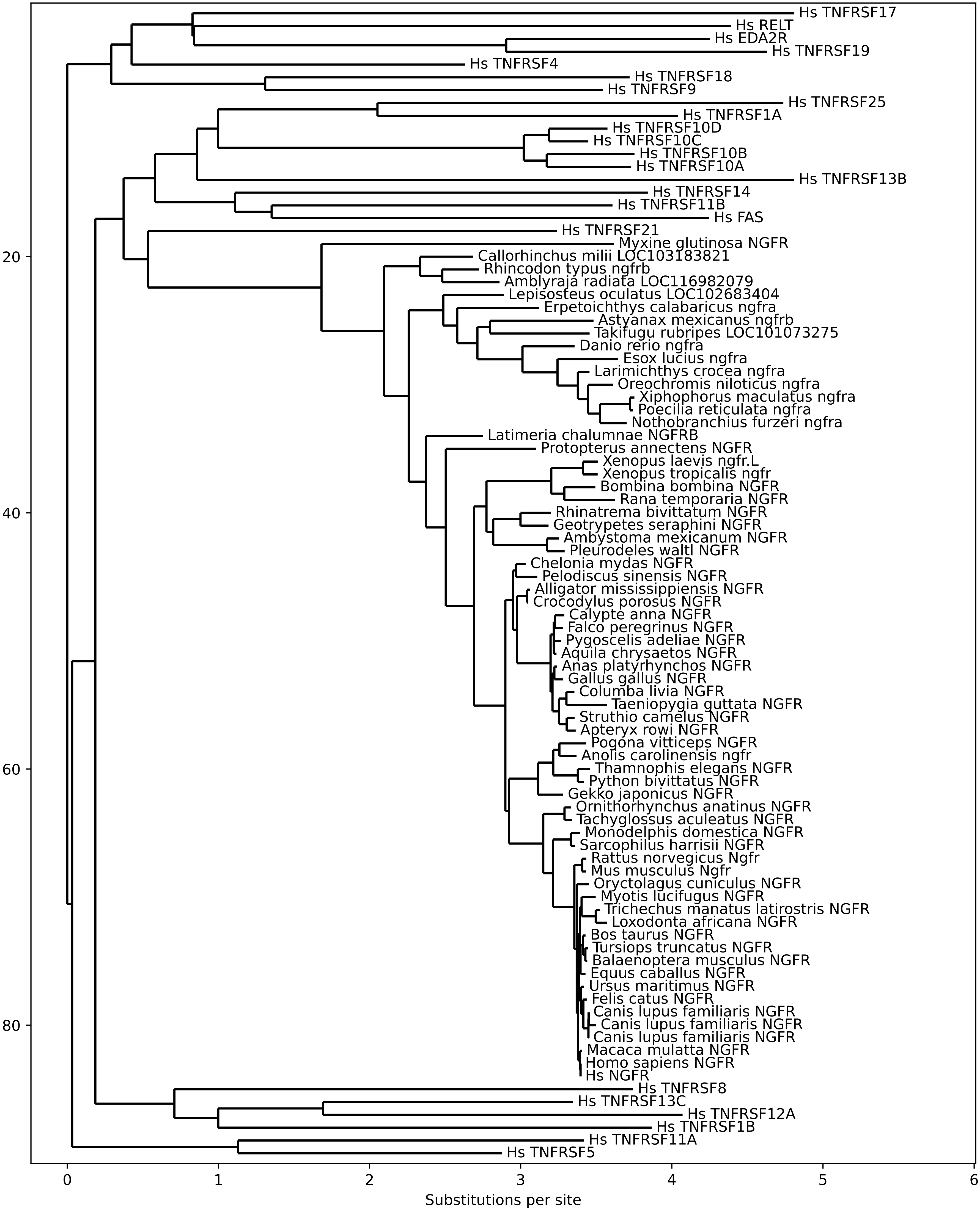

Supplement: Supplementary file 1 [file biology-15-01695-s001.zip › Supplementary_Figure_S1.pdf]
